# Role of depression in secondary prevention of Chinese coronary heart disease patients receiving percutaneous coronary intervention

**DOI:** 10.1371/journal.pone.0187016

**Published:** 2017-12-21

**Authors:** Can Feng, Tao Ji, Yu Liu, Danfeng Chen, Jia Dai, Xiushi Ni, Jiaqi Zhu, Xuebo Liu, Xianxian Zhao

**Affiliations:** 1 Yueyang Hospital of Integrated Traditional Chinese and Western Medicine, Shanghai University of Traditional Chinese Medicine, Shanghai, China; 2 Department of Neurosurgery, The Tenth Affiliated Hospital, Tongji University, Shanghai, China; 3 Department of Cardiology, The Gulou Hospital attached to Nanjing University, Nanjing, China; 4 Department of Cardiology, Changhai Hospital, Second Military Medical University, Shanghai, China; 5 Department of Geriatrics, Shanghai First People's Hospital Affiliated Shanghai Jiaotong University, Shanghai, China; 6 Department of Cardiology, Shanghai Tongji Hospital, Tongji University School of Medicine, Shanghai, China; Osaka University Graduate School of Medicine, JAPAN

## Abstract

**Introduction:**

Coronary heart disease (CHD) patients who have undergone percutaneous coronary intervention (PCI) have higher rates of depression than the general population. However, few researchers have assessed the impact of depression on the secondary prevention of CHD in China.

**Objective:**

The main purpose of this investigation was to explore the relationship between depression and secondary prevention of CHD in Chinese patients after PCI.

**Methods:**

This descriptive, cross-sectional one-site study recruited both elective and emergency PCI patients one year after discharge. Data from 1934 patients were collected in the clinic using questionnaires and medical history records between August 2013 and September 2015. Depression was evaluated by the 9-item Patient Health Questionnaire. Secondary prevention of CHD was compared between depression and non-depression groups.

**Results:**

We found that depression affected secondary prevention of CHD in the following aspects: lipid levels, blood glucose levels, smoking status, physical activity, BMI, and rates of medication use.

**Conclusions:**

Depressive patients with CHD are at increased risk of not achieving the lifestyle and risk factor control goals recommended in the 2006 AHA guidelines. Screening should focus on patients after PCI because treating depression can improve outcomes by improving secondary prevention of CHD.

## Introduction

Coronary heart disease (CHD) is the leading cause of morbidity and mortality globally, and when combined with stroke, it accounted for 17.5 million deaths in 2012[1]. One of the most commonly used treatments for CHD is percutaneous coronary intervention (PCI), which has become more commonly used than coronary artery bypass graft [2], as PCI is a safe, efficient and less costly revascularization procedure[3]. Approximately 400,000 CHD patients undergo PCI in China every year. There are some procedure-related psychological reactions after PCI. Notably, depression is significantly correlated with adverse cardiac events in CHD[4]. The prevalence of depression in patients with acute myocardial infarction has been reported to be approximately 20%[5]. A previous investigation indicated that patients who are depressed after suffering from an acute coronary syndrome have poor cardiac outcomes and an increased risk of mortality after accounting for risk factors[6]. Some research has indicated that depression is correlated with a significant increase in the risk of negative health outcomes in patients experiencing coronary revascularization, independent of traditional risk factors[6] [7] [8]. CHD patients who have depression are at an increased risk for recurring cardiac events after PCI[9]. Depression typically comprises manifestations such as a sense of depressed emotion, a loss of affection or enjoyment in activities, sleep disorders, fatigue, and diminished concentration. Depression can significantly decrease engagement in lifestyle modifications that are essential to halting the progression of CHD. Secondary prevention programs are known to be essential to decreasing the burden of progression in CHD. Furthermore, the main modifiable risk factors affecting the development and progression of CHD are smoking, hypercholesterolemia, overweight and obesity, physical inactivity, hypertension, and diabetes[10], all of which may be affected by depression. Therefore, the objective of this investigation is to explore this relationship between depression and secondary prevention of CHD in patients who have undergone PCI. We chose one year after PCI as the timeframe of our investigation because we wanted to determine the prevalence of depression and the implementation of secondary prevention methods after one year of recovery.

## Methods

### Study design

The present investigation was a descriptive and cross-sectional survey using a structured questionnaire and medical history including laboratory tests of CHD patients. The participants were recruited consecutively from a coronary follow-up clinic between August 2013 and September 2015 at their one-year follow-up after PCI. The reason for the one-year time point is because in our hospital, as in many hospitals in China, post-PCI patients are asked to return to the clinic for a comprehensive examination 1 year after the procedure. This follow-up visit is an easy way to obtain patients’ data and clinical condition. We therefore chose this time point to attain the cross-sectional information in this study. After obtaining informed consent, patients’ eligibility was confirmed by analyzing their medical records for the inclusion and exclusion criteria. Participants were included if they (1) were 18 to 70 years old; (2) had a diagnosis of coronary heart disease; (3) underwent PCI one year ago; (4) accepted participation in this study; and (5) were able to speak, read, and write Chinese. Participants were excluded if they had (1) a terminal illness, (2) abnormal renal and liver function, (3) a limb deficiency, or (4) a language comprehension disorder.

### Instruments and measurements

Two investigators (Feng C. and Ji T.) interviewed the patients to gather sociodemographics such as age, gender, type of PCI, education, cigarette smoking status, body mass index, hypertension history, diabetes mellitus, and self-management abilities (physical activity). Data from physical examinations [including height, weight, and systolic and diastolic blood pressure (SBP and DBP, respectively)] and biochemical testing [total cholesterol (TCHO), total triglycerides (TG), low-density lipoprotein cholesterol, high-density lipoprotein cholesterol, fasting blood glucose (FBG), and glycated hemoglobin A1c (HbA1c) levels] were obtained from all participants. These medial data was obtained from their medical chart in our medical records system. Participants’ depression status was calculated via the 9-item Patient Health Questionnaire (PHQ-9), which was administered in a private room using structured questionnaires. The PHQ-9 consists of nine items, each of which assesses the existence of 1 of the 9 DSM-IV criteria for a depressive episode in the past two weeks. Each question in the PHQ-9 is answered using a 4-point scale ranging from 0 (never) to 3 (nearly every day), for a total score ranging from 0 to 27; higher scores indicate a higher likelihood of major depressive disorder. The PHQ-9 questionnaire is a one-page survey and can be accomplished alone. The PHQ-9 questionnaire was first translated into Chinese by a bilingual psychiatrist. The answers were reviewed by 2 independent research coordinators for accuracy.

### Validation of the PHQ-9 in the Chinese sample

The translated version was back-translated and modified until the back-translated version was comparable with the original English version. Some patients were invited to review the Chinese version and to provide feedback. Some modifications were made before the final version of the PHQ-9 was completed. The reliability of the Chinese version of the PHQ-9 was tested. The internal consistency value, obtained by using Cronbach α coefficient, was 0.81 (95% CI, 0.80–0.83). To assess test-retest reliability, 265 patients completed the PHQ-9 a second time within 2 weeks. The intraclass correlation coefficient for test-retest reliability of the total scores was 0.86 (95% CI, 0.83–0.91; F = 7.73, df = 264, P<0.01), demonstrating limited variability between the two-week time points.

### Measurement of physical examination and biochemical variables

After the participants had rested for 10 min, blood pressure (BP) was obtained three times with a desktop mercury column sphygmomanometer with participants in a seated position. The time interval between each measurement was 2 minutes. The average of the BP values was calculated and used for analysis. Blood samples were drawn from each patient after they had fasted for at least 12 h and rested overnight. FBG levels were obtained using oxygen electrodes; TCHO levels were measured using the cholesterol oxidase method; TG levels were measured using the enzymatic method; and HDL-C and LDL-C levels were directly measured using the clearance method. GHbA1c level was measured using high-performance liquid chromatography.

### Definitions and outcomes

In the PHQ-9, compared with a lower score, a higher score reveals more depression. As indicated previously, a score of 10 is the ideal cutoff for detecting the presence of major depression in Chinese patients [11]. We therefore used the cutoff value of ≥10 for major depression. The goals of secondary prevention of CHD include the following: 1) complete non-smoking: never smoked or stopped smoking for at least 3 months; 2) ≥30 minutes of moderate-intensity aerobic activity per day ≥5 days per week: patients self-reported their physical activity mode and duration; 3) weight management resulting in BMI >18.5 kg/m^2^ and <25.0 kg/m^2^; 4) BP<140/90 mm Hg; 5) FBG <6.11 mmol/L in DM patients, and 6) LDL-C<2.6 mmol/L.

### Ethical considerations

This investigation was approved by the ethics committee of Shanghai Changhai Hospital before subject enrollment, and it adhered to the principles of the Declaration of Helsinki (as revised in Brazil 2013). All participants in this research read and signed an informed consent.

### Statistical analyses

For the statistical analyses of the data in this study, the Statistical Package for the Social Sciences (SPSS) version 22 (IBM Corp, Armonk, New York) was used. Differences between continuous variables were evaluated using t-tests, and the χ^2^ test was used for categorical variables. Logistic regression analyses were used to evaluate the associations between depression and secondary prevention of CHD patients after PCI by calculating adjusted odds ratios (ORs) and 95% confidence intervals (CIs). Adjusted factors included type of PCI, education, and amount of smoking (cigarettes per day). Missing data were not imputed. The significance level was set at .05. All demographic and clinical data, with the exception of age, are reported as frequencies and percentages; age is reported as the mean and standard deviation. Descriptive statistics, mean T standard deviations, or percentages were used to describe the participant profiles.

## Results

### Population characteristics

Of the 1934 patients enrolled, 30 (1.5%) subjects were excluded due to incomplete or missing data; these patients were missing at random. A total of 756 patients were female, the mean age ± standard deviation (SD) was 55.64 ±10.6 years (range, 31–76 years), and BMI was 27.3±2.3. In this study, 52.32% of patients had hypertension, and approximately 31.08% of patients had DM. Nearly a quarter of patients received emergency PCI. Most of the patients had attained less than a college education. The non-smoking rate was nearly 70%. As evaluated by the PHQ-9, the average depression score was 8.54±3.41. Using the cutoff score of ≥10, 267 (13.8%) of the PCI patients were determined to be depressed. Sociodemographic and clinical characteristics are shown in Table 1.

**Table 1:** The sociodemographic and clinical characteristics of the study population before the PCI.

Patient Characteristics Mean±SD, n(%)	Depression	Non-depression	*P*
**Age, y**	56.4±9.7	54.4±11.3	0.11^a^
**Female**	98(13%)	658(87%)	0.39^b^
**Male**	169(14.3%)	1009(85.7%)	
**Type of PCI**	189(12.7%)		0.009^b^
** elective**	78(17.5%)	1300(87.3%)	
** emergency**		367(82.5%)	
**Education**	187(12.7%)		<0.001^b^
** Lower than middle school**	53(12.9%)	1283(87.3%)	
** High school**	27(50.9%)	358(87.1%)	
** More than college**		26(49.1%)	
**Amount of smoking (cigarettes per day)**			0.024^b^
** None**	180(13.4%)	1164(86.6%)	
** ≤10**	35(20.1%)	139(79.9%)	
**≥10, ≤20**	30(15.5%)	163(84.5%)	
** ≥20**	22(9.9%)	201(90.1%)	
**Body mass index, kg/m**^**2**^	26.9±2.1	27.5±3.3	0.24^a^
**history of HTN**	134(50.2%)	878(52.7%)	0.451^b^
**DM**	85(31.8%)	584(35%)	0.308^b^
**TCHO, mmol/L**	4.53±0.87	4.24±1.22	0.12^a^
**TG, mmol/L**	1.67±0.78	1.63±0.77	0.24^a^
**LDL-C, mmol/L**	2.81±0.64	2.77±0.24	0.22^a^
**HDL-C, mmol/L**	1.01±0.27	1.10±0.16	0.08^a^

Abbreviations: SD, standard deviation; PCI, percutaneous coronary intervention; HTN, hypertension; DM, diabetes mellitus (Note: The pre-PCI depression data were not available, and the grouping of data by depression status was made solely based on the one-year scores)

^a^P-values was based on t-tests.

^b^P-values were based on χ^2^ test.

### The influence of depression on secondary prevention: Bivariate analysis

Patients were divided by depression status into two groups. Comparisons are shown in Table 2. FBG, HbA1c, TCHO, TG, LDL-C, and HDL-C were significantly different between the depression and non-depression groups, while BMI, SBP, and DBP were not. For rate of control of risk factors, smoking quit rate, physical activity, BMI, lipids, and glucose levels were statistically significantly different between groups, while BP was not. Medication use was also shown in Table 2. The usage rates of angiotensin converting enzyme inhibitor (ACEI) / angiotensin receptor blocker (ARB)s,β-blockers, and lipid-lowering drugs were significantly lower in the depression group.

**Table 2:** Difference between the depression group and the non-depression group in the secondary prevention of CHD.

Variable	Depression	Non-depression	*P*
**BMI, kg/m**^**2**^	26.92±3.42	27.02±4.34	0.755
**FBG, mmol/L**	6.32±1.64	5.52±1.81	<0.001
**HbA1c, %**	8.6±1.1	6.3±0.8	0.034
**TCHO, mmol/L**	4.47±1.11	3.80±0.86	<0.001
**TG, mmol/L**	1.56±0.92	1.43±0.81	<0.001
**LDL-C, mmol/L**	2.72±0.74	2.02±0.34	<0.001
**HDL-C, mmol/L**	0.98±0.28	1.10±0.31	<0.001
**Control rate of risk factors, n(%)**			
**Smoking quit rate**	52(19.5)	1000(60)	<0.001
**Physical activity**	81(30.4)	1130(67.8)	<0.001
**BMI**	103(38.5)	763(45.8)	<0.001
**BP**	200(75)	1284(77)	0.067
**Lipid**	162(60.7)	1460(87.6)	<0.001
**Glucose**	102(60.8)	1174(70.4)	<0.001
**Medication use rate, %**			
**ACEI/ARB**	151(56.7)	1135(68.1)	0.014
**β-blocker**	209(78.4)	1457(87.4)	0.045
**Antiplatelet**	255(95.3)	1579(94.7)	0.7
**Lipid-lowering drug**	181(67.8)	1512(90.7)	<0.001

Data are expressed as the means ± SD. BMI, body mass index; FBG, fasting blood glucose; GHbA1c, glycated hemoglobin A1c; TCHO, total cholesterol; TG, triglycerides; LDL-C, low-density lipoprotein cholesterol; HDL-C, high-density lipoprotein cholesterol; BP, blood pressure; ACEI/ARB, angiotensin-converting enzyme inhibitor/angiotensin-receptor blocker

### The influence of depression on secondary prevention: Multivariate analysis

Considering the significant differences in factors such as type of PCI, education, and amount of smoking (cigarettes per day) at baseline, we conducted logistic regression analyses to control for the baseline impact. We defined depression state as the dependent variable (depression: 0; non-depression: 1), while type of PCI, education, amount of smoking (cigarettes per day), smoking quit rate, physical activity, BMI, lipids, glucose, ACEI/ARBs, β-blockers, and lipid-lowering drugs were independent variables. Table 3 shows that smoking quit rate, physical activity, BMI, lipids, glucose, lipid-lowering drugs and education were factors that were strongly related to depression after adjusted the factor of type of PCI, amount of smoking, ACEI/ARB and β-blocker.(P<0.05).

**Table 3:** Multiple regression analysis of factors connected with depression in post-percutaneous coronary intervention patients.

Variable	*β*	Wald *χ*^2^	*P*	*OR*
**Type of PCI**	-0.12	2.41	0.12	0.89(0.77–1.03)
**Education**	-0.02	5.30	0.02	0.98(0.96–0.99)
**Amount of smoking (cigarettes per day)**	-0.11	0.60	0.44	0.90(0.69–1.17)
**Smoking quit rate**	0.64	7.62	0.006	1.92(1.20–3.02)
**Physical activity**	1.78	6.38	0.012	5.92(1.48–23.52)
**BMI**	1.36	6.74	0.009	3.88(1.40–10.84)
**Lipids**	1.42	4.30	0.038	4.16(1.08–16.08)
**Glucose**	1.90	4.18	0.041	6.66(1.08–41.10)
**ACEI/ARB**	0.62	0.76	0.380	1.85(0.46–7.39)
**β-blocker**	0.35	0.30	0.587	1.41(0.41–4.89)
**Lipid-lowering drug**	2.00	6.03	0.017	7.41(1.50–36.67)

## Discussion

Depression is frequently encountered as a response to an acute coronary episode or a correlated procedure such as PCI; in our study, the PCI population sample had a rate of depression of 13.8%, which is much higher than that of the normal population (3.6%)[12]. It is worth noting that the prevalence of depression in the CHD patients in our study differs from that of previous studies (34.6% to 45.8%) [13]. First, the cutoff point (≥10) may mainly account for this difference, as some researchers use 5 as the cutoff score in the PHQ-9. Second, one year may be enough time for the patients to recover from depression after PCI. Third, there may be some patients who cannot or choose not to answer the questionnaire honestly. As several studies have previously illustrated, in China, people with depression typically do not want to admit to being depressed out of fear of being labeled insane, which may also contribute to the difference in prevalence from previous studies.

To the best of our knowledge, our study is the first to identify the relationship between the secondary prevention of CHD in patients after PCI and depression. In our study, there was a substantial number of patients who did not implement the lifestyle modifications and risk factor control objectives recommended in the 2006 AHA guidelines[14]. Patients with CHD who have depression are at an increased risk of not achieving the lifestyle and risk factor control goals outlined for secondary prevention. In our study, after controlling for differences in the baseline data, we found that depressed patients reported lower medication compliance (lipid-lowering drugs), lower smoking quit rates, poor control of BMI, higher lipid and glucose levels, and lower levels of physical activity. These aspects are all vital to halting the progression of coronary heart disease. Physicians should pay more attention to and be alert for depressive symptoms accroding to our findings, since depression status also increases the risk of cardiovascular disease, possibly due to its potential biological mechanisms involving pro-inflammatory cytokines, worsening endothelial function, and coagulation factors[15].

There are several possible suggestions to tackle this issue. First, we suggest that physicians should pay more attention to patients with depression and encourage them to seek help from a mental health professional. After the interview, we informed the patients in our study who were considered to have depression, but they demonstrated a low understanding of the basic facts of depression, and most of them were not interested in speaking about depression. This phenomenon has been reported before and is one of the reasons why most patients with mental disorders do not seek professional consultations and are therefore left to cope on their own[16]. One possible interpretation of this finding is that fear of stigmatization might prompt Chinese individuals to be much more likely to deny being depresssed. Second, most cardiologists renounce their responsibility in confirming that depression status is evaluated. Currently, a large proportion of cardiologists in China do not believe that they should evaluate their patients for depression and think that it is the duty of a nurse or the family physician. Although nurses may provide psychoeducational education to help reduce depression, doctors should join in the management of cardiovascular risk factors by diagnosing and treating depression.

For depressed patients who have undergone PCI, the management strategies are similar to those of other depressed patients and include exercise programs, cognitive behavioral therapy (CBT), general support, and antidepressant medication. Exercise programs seem to be very effective at reducing depression[17]. General support from family members and friends is also very essential to depressed patients. Therefore, it may be valuable to include significant others in the education of the patients. In addition to psychological consultations and rehabilitation, antidepressant medication is also effective in the treatment of depression. Treatment for depression among cardiac patients is effective for improving depressive symptoms and may improve cardiac mortality[18] [19]. However, there are issues concerning potential risks of medication. One of the major potential hazards of antidepressant medications is their impact on prolonging cardiac myocyte action potentials. This is especially the case for tricyclic antidepressants, and thus when prescribing medication for depression, physicians should consider and monitor for this side effect. It is notworthing that selective serotonin reuptake inhibitors (SSRIs) have been found to have a protective cardiovascular effect. They may be suggested for use rather than tricyclics.

There are some limitations in our research. First, pre-PCI depression data are not available, and this is a major limitation. Second, the 1-year study time point should also be taken into consideration, because it is quite a long time after the PCI. Third, there are no other data points more proximal to the procedure to assess the effect of PCI. In addition, this is a single-site study, which is always a limitation to the generalizability of findings. Finally, in the area of secondary prevention, dietary control was omitted because it cannot be clearly demonstrated by most of the participants.

Depression is common in CHD patients and is correlated with higher mortality and morbidity rates. In our study, we found that depression has a strong impact on several approaches to secondary prevention in CHD patients. Although more investigations are needed to clearly and consistently establish the cardiovascular impact, depression itself affects quality of life. More importantly, it reduces compliance in adherence to medical and lifestyle strategies. There is significant research supporting the initiation of exercise programs, general support, and antidepressant medications to reduce depression in the CHD population.

## Supporting information

S1 Table STROBE statement—Checklist of items that should be included in reports of observational studies. (DOC)
